# G-Causality Brain Connectivity Differences of Finger Movements between Motor Execution and Motor Imagery

**DOI:** 10.1155/2019/5068283

**Published:** 2019-10-02

**Authors:** Chao Chen, Jiaxin Zhang, Abdelkader Nasreddine Belkacem, Shanting Zhang, Rui Xu, Bin Hao, Qiang Gao, Duk Shin, Changming Wang, Dong Ming

**Affiliations:** ^1^Key Laboratory of Complex System Control Theory and Application, Tianjin University of Technology, Tianjin 300384, China; ^2^Academy of Medical Engineering and Translational Medicine, Tianjin University, Tianjin 300072, China; ^3^Department of Computer and Network Engineering, College of Information Technology, UAE University, P.O. Box 15551, Al Ain, UAE; ^4^Zhonghuan Information College Tianjin University of Technology, Tianjin 300380, China; ^5^Department of Electronics and Mechatronics, Tokyo Polytechnic University, Kanagawa 243-0297, Japan; ^6^Beijing Key Laboratory of Mental Disorders, Beijing Anding Hospital, Capital Medical University, Beijing 100088, China; ^7^North China University of Science and Technology, Tangshan, Hebei 063000, China

## Abstract

Motor imagery is one of the classical paradigms which have been used in brain-computer interface and motor function recovery. Finger movement-based motor execution is a complex biomechanical architecture and a crucial task for establishing most complicated and natural activities in daily life. Some patients may suffer from alternating hemiplegia after brain stroke and lose their ability of motor execution. Fortunately, the ability of motor imagery might be preserved independently and worked as a backdoor for motor function recovery. The efficacy of motor imagery for achieving significant recovery for the motor cortex after brain stroke is still an open question. In this study, we designed a new paradigm to investigate the neural mechanism of thirty finger movements in two scenarios: motor execution and motor imagery. Eleven healthy participants performed or imagined thirty hand gestures twice based on left and right finger movements. The electroencephalogram (EEG) signal for each subject during sixty trials left and right finger motor execution and imagery were recorded during our proposed experimental paradigm. The Granger causality (G-causality) analysis method was employed to analyze the brain connectivity and its strength between contralateral premotor, motor, and sensorimotor areas. Highest numbers for G-causality trials of 37 ± 7.3, 35.5 ± 8.8, 36.3 ± 10.3, and 39.2 ± 9.0 and lowest Granger causality coefficients of 9.1 ± 3.2, 10.9 ± 3.7, 13.2 ± 0.6, and 13.4 ± 0.6 were achieved from the premotor to motor area during execution/imagination tasks of right and left finger movements, respectively. These results provided a new insight into motor execution and motor imagery based on hand gestures, which might be useful to build a new biomarker of finger motor recovery for partially or even completely plegic patients. Furthermore, a significant difference of the G-causality trial number was observed during left finger execution/imagery and right finger imagery, but it was not observed during the right finger execution phase. Significant difference of the G-causality coefficient was observed during left finger execution and imagery, but it was not observed during right finger execution and imagery phases. These results suggested that different MI-based brain motor function recovery strategies should be taken for right-hand and left-hand patients after brain stroke.

## 1. Introduction

EEG-based brain-computer interfaces (BCIs) have been used for building an advanced communication or control pathway between brain and computer using noninvasive measurements [1, 2]. Several classical BCI paradigms were developed for helping handicapped people to interact with the environment by controlling a smart home, robotic arm, and a wheelchair using brain activity based on event-related potential (ERP) such as the P300 wave [3, 4] or based on steady-state visually evoked potential (SSVEP) [5] and motor imagery [6, 7].

The mental process during motor execution or motor imagery has been widely used for building BCI systems in several domains [8–10]. This mental process has also showed the potential applications in the rehabilitation field for patients who suffered from brain strokes [11, 12]. These patients who lost some motor functions after brain stroke might be able to reactivate some brain areas such as sensorimotor area [13] by using BCI based on motor imagery as one of the most effective surrogate motor training methods [14]. When patients perform or imagine hand gestures or finger movements during rehabilitation exercise sessions, they activate more areas of the brain and therefore maximize the neuroplastic benefits. These kinds of movement require a greater overall activation of muscle contraction and therefore probably require the firing of a greater number of cortical cells. Furthermore, the sophisticated movements of fingers are important to accomplish many complex tasks in daily life. Unfortunately, the patient who suffered contralateral hemiparesis after brain stroke lose the ability of moving their fingers which means they lose the ability of motor execution [15, 16]. Stroke is a kind of impaired cerebrovascular diseases. The number of patients who suffered from this disease in recent years was about 2 million per year, age standardized incidence is about 21/6250, and 70%∼80% of stroke patients have different degrees of movement disorders. Based on the motion, performing physical therapy is helpful for the recovery of motor function in patients with cerebral apoplexy but requires patients to have a certain ability of independent movement. Most stroke patients have poor exercise ability in the early rehabilitation stage, during which exercise imagination therapy can play an important role [17, 18]. However, the ability of motor imagery might be a useful option for restoring and recovering the motor functions [19, 20]. Which kind of motor imagery strategy is more suitable according to the patients' condition, e.g., subject-dependent recovery protocol, and which kind of feature is more suitable to evaluate subject-dependent motor function can be evaluated and can be employed to build a neurofeedback system to improve the motor recovery protocol. Whether the brain connectivity can be used as an effective feature for motor function evaluation and motor recovery is found. The efficacy of motor imagery for neural prosthetics control and motor recovery after brain stroke is still very much an open question.

Beta-band activities play an important role in motor imagery. In previous research papers, researchers noticed that event-related desynchronization occurs during left hand, right hand, foot, and tongue motor imagery tasks [21–23]. They showed a significant usage of beta-band activities for motor recovery in stroke patients [17–24].

Brain function is increasingly understood to be a result of extensively interconnected neurons which means the brain connection reflects the brain function such as decision-making [25–27] and motor function recovery [28–31]. Asymmetry also exists in the perspective of functional connectivity [32]. It has been shown that right-handed subjects who completed the motor imagination tasks with the right hand had more effective connections between the auxiliary motor area and other brain regions than those who completed the motor imagination tasks with the left hand and so on. This lateralization of the cerebral cortex may be related to the asymmetry of the brain structure of right-handed subjects [33].

To investigate the brain connectivity during finger motor execution and motor imagery tasks, a new EEG experimental paradigm using thirty finger gestures was designed. Right-handed subjects only participated in the proposed experiment. First, they were instructed to watch a short video and mimic the finger movements by performing the movements. Second, they were asked to imagine the finger movements for sixty trials for each movement. However, we investigated beta-band activity of EEG signals which was recorded and extracted from both scenarios: motor execution and motor imagery. Also, the Granger causality (G-causality) analysis method was applied to calculate the brain network between contralateral primary motor area, premotor area, and primary sensory area. G-causality coefficients of motor execution and imagery under left-hand and right-hand conditions were computed and analyzed. G-causality is the most adopted criterion for causal inference in brain recordings knowing that the number of G-causality over many trials or observation epochs means how many times statistically significant brain connection was built and the coefficients of G-causality indicate the strength of the brain connections. Using these G-causality characteristics, we were able to investigate the efficacy of motor imagery of finger movements using noninvasive measurements and compare it with motor execution.

## 2. Method

### 2.1. Data Recording and Experimental Paradigm

**Figure 1:**
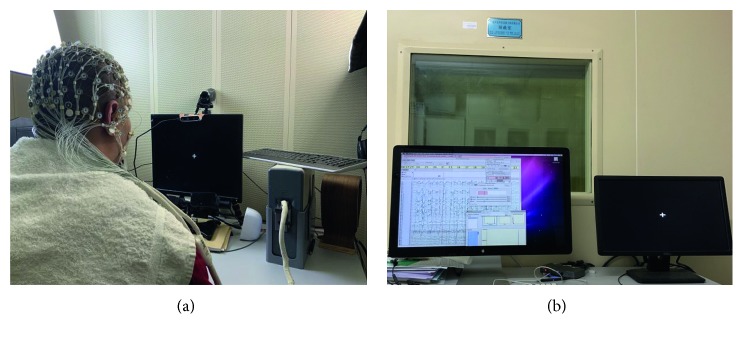
Experiment setup of real-time recording of EEG signals during performing or imagining finger movements. We can see in this picture, two subfigures from left to right: the experimental electromagnetic shielding room and the data collection and analysis platform Net Station (i.e., real-time streaming, recording, and some preprocessing of multichannel EEG data) and experimental paradigm interface.

For reducing signal interferences, EEG experiments were held in the electromagnetic shielding room (Figure 1) at Beijing Anding Hospital, Capital Medical University, China. This study was approved by the Ethics Committee of North China University of Science and Technology, Hebei Province, China (Number: 2019002). All participants provided written informed consent. Eleven healthy people (7 males and 4 females) were recruited to participate in this study. All of them are right-handed and have no experience in motor execution and/or motor imagery EEG-based BCI experiments. Chirality is determined by the Edinburgh handedness inventory (EHI). EHI produces scores ranging from −100 (strongly left handed) to 0 (unbiased handedness) and 100 (strongly right handed). The average score of handedness of the subjects is 90.84, and the standard deviation is 5.79. The average age of them is 25 years (the range is from 22 to 27).

The subjects were instructed to sit down on a comfortable chair in front of the computer screen which was about 50 cm away from their eyes. Before the experiment, a clear explanation of the framework of experimental paradigm was given to the subjects. The experimental paradigm was designed by using *E-prime* which is a software package used to design and run simulation experiments, with a focus on psychological and cognitive science.

The 128 EEG channels were recorded during two scenarios: motor execution and motor imagery of finger movements by using EGI signal acquisition system (Brain products, Germany). The sampling rate was 1000 Hz, where oversampling produced no significant changes in timing and amplitude. As recording setting, EEG signals were band passed from 0.1 Hz to 50 Hz and saved with the Net Station system, as shown in Figure 1. We removed high frequency bands such as high and low gamma bands because it is hard to get them in a single trial using noninvasive measurements such as EEG.

In the beginning of each session, a clear guideline of the proposed experiment was shown in the screen to guide the subjects and make them more familiar with the experimental paradigm process. The subjects were able to understand fully the experimental paradigm and complete the whole instructions more attentively. The subjects could take few minutes' rest after each session.

**Figure 2:**
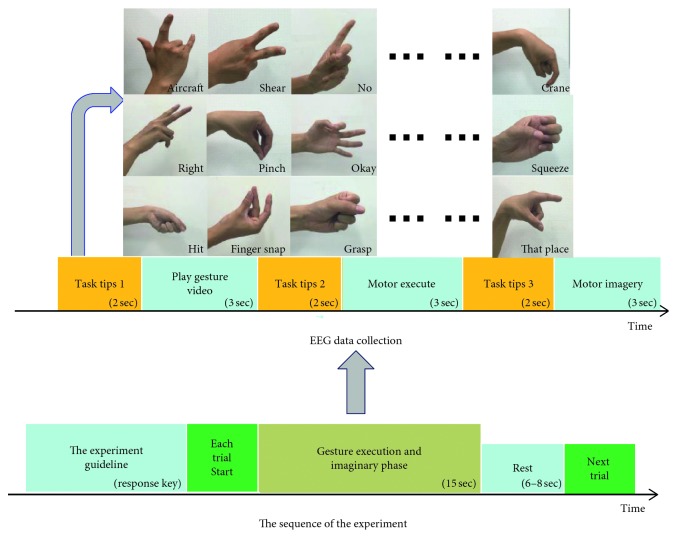
The experimental paradigm. The gesture execution and imaginary phase contains three task tips and three duration of playing gesture video (3 seconds), motor execution, and imagery (2 seconds), in which each of thirty finger movements were used. Thirty finger movements are okay, no, begun to refer, phase modulation, crane, right, scope, aircraft, geometry, crowded, scissors, cut, there, kneading, clap, planer, money, broom, fan, double, frost, question marks, grip, rain, circuitous, approximately equal to, blame, recruit, at noon, and catch.

For each trial, the finger gestures during execution and imagery phases were arranged within 15 s, containing three instructions or tips of 2 seconds for each task, gesture video of 3 s, finger motor execution of 3 s, and motor imagery of 3 s. Task tip 1 indicates that the video will start. One of thirty finger movements was shown randomly during the gesture video time. Task tips 2 and 3 indicated the subjects to perform or imagine the same finger movement at motor execution and motor imagery phases, respectively. Then, the subject can take a rest at a duration from 6 seconds to 8 seconds. Then, the next trial will be conducted (Figure 2).

### 2.2. Brain Region of Interest Selection

**Figure 3:**
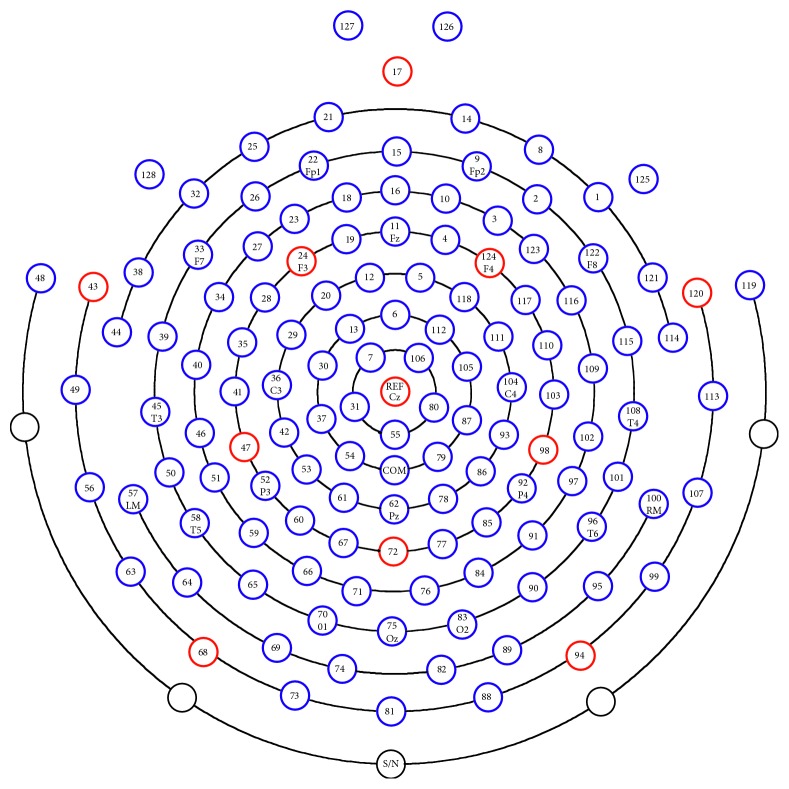
The EEG electrode position of 128 channels. Using the EGI signal acquisition system Net Station (Brain product, Germany), 128 EEG channels were recorded.

In this research, the difference between motor execution and motor imagery was investigated. The premotor area, primary motor area, and primary sensory area might be the most relevant brain areas during performing and imagining a finger movement. The position of 128 channels is shown in Figure 3. Due to the low spatial resolution of EEG signals, two electrodes E13 and E20 and E112 and E118 were averaged to calculate the brain activities of the left and right premotor area, respectively, according to location of the premotor area in previous EEG-TMS research [34]. The electrodes E36 correspond to the left motor areas. The electrodes E104 correspond to the primary right motor areas. The electrodes E52 correspond to the left primary sensory areas. The electrodes E92 correspond to the right primary sensory areas.

### 2.3. EEG Data Processing

The EEGLab toolbox [35] was used for the preprocessing phase of raw EEG data. In this phase, we have checked the quality of EEG signals by checking the signal-to-noise ratio. Then, the bad channels with obvious artifacts (e.g., clear muscles artifact or strong blink) were removed using EEGLab functions and were not included in results analysis. However, few trials only were not recorded correctly. In our proposed experiment, 60 trials of EEG signals for each subject were recorded for left and right finger movements during motor execution and motor imagery phases. The numbers of valid trials of each subject are shown in Table 1.

**Figure 4:**
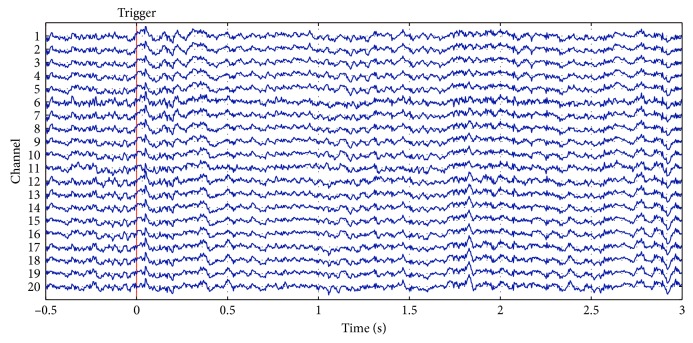
Representative EEG signals before baseline drift. There is a total of 128 channels of data. The figure shows the EEG waveform of 1–20 channels.

**Figure 5:**
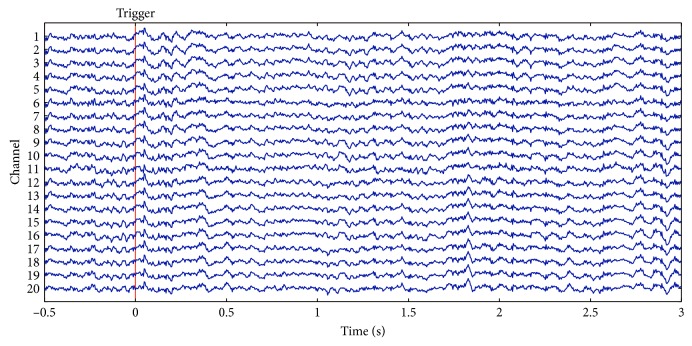
EEG signals after baseline drift. There is a total of 128 channels of data. The figure shows the EEG waveform of 1–20 channels.

The 128 EEG signals were down sampled from 1 KHz to 500 Hz for reducing data size. Then, the baseline of EEG signals was reset called baseline drift [36]. There are several methods to remove baseline drift, such as the median filtering method [37], wavelet transform method [38], high-pass filtering method [39, 40], and curve fitting method [41]. In this paper, weighted least squares- (WLS-) [42] based local linear regression method is employed to fit the original data of each segment to zero. Figures 4 and 5 show EEG signals before and after removing baseline drift, respectively.

**Figure 6:**
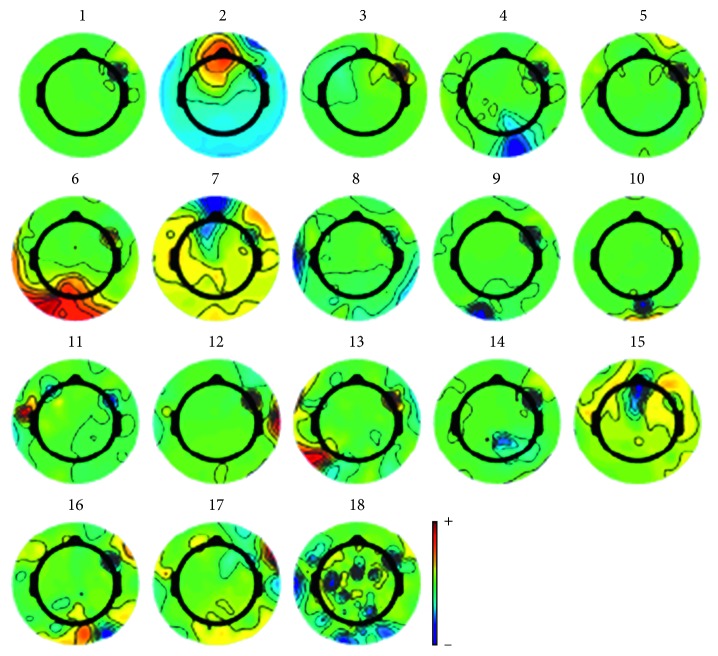
Spatial distribution map of ICA decomposition components. After ICA processing, 18 independent components are obtained, which can be seen as eye electrical components from the second component.

The ICA method was used to remove the artifact in EEG signals such as eye movements (EOG signal) per session. At first, EEG signals were decomposed by ICA, and the limited number of components was determined through the whitening stage of PCA. The ICA decomposed components in spatial distribution map of each EEG signal are shown in Figure 6, respectively. It was clear that the energy of the second component was mainly concentrated in the area around the eyes, which was consistent with the EOG signal area in the prefrontal cortex. Thus, artifact of the eye movement can be removed from EEG signals by deleting the second component.

After removing the eye movements' artifacts, the beta-band activities (14–30 Hz) during −0.5 to 3 second periods of the motor execution and imagery onset were feature selected for Granger causality brain connectivity calculation.

### 2.4. Granger Causality Brain Connectivity Calculation

The Granger causality analysis model is based on the autoregressive model, which depends on the time priority of signals and can be used to measure the degree of mutual influence between signals. And it can be used to explore the temporal relationship between regions of interest in order to reveal the directional information flow between brain regions. The Granger causality analysis model was already used for computing the brain connection of decision-making and motor recovery [25–29], which showed that the Granger causality analysis is effective for analyzing the brain.

Given the two wide-sense stationary time series *X* and *Y*, which have constant means and variances. If the predictive effect of the variable *X* by using the past information of *X* and *Y* is better than the predictive effect using the past information of *X* significantly, then the variable *X* is the Granger cause by the variable *Y* [43]. The autoregressive model of *X* can be calculated by the following equation.

Constrained regression model:

(1) $$\begin{array}{c} X_{t}=\alpha_{0}+\overset{p}{\underset{i=1}{\sum }}\alpha_{i}X_{t-i}+\varepsilon_{x}. \end{array}$$

Unconstrained regression mode (*u*):

(2) $$\begin{array}{c} X_{t}=\alpha_{0}+\overset{p}{\underset{i=1}{\sum }}\alpha_{i}X_{t-i}+\overset{q}{\underset{i=1}{\sum }}\beta_{i}Y_{t-i}+\varepsilon_{y}, \end{array}$$

where *α*_0_ represents the constant term; *p* and *q* are the maximum lag number of variables *X* and *Y*, respectively; and *ε*_*x*_ and *ε*_*y*_ denoted residuals of constrained and unconstrained regression models, respectively. The Bayesian information criterion (BIC), Akaike information criterion (AIC), and experiential method are usually used to calculate the lag number of the regression model. In the study, the max lag of *X* and *Y* was set to 20.

The sum of squared residuals of constrained regression models can be calculated by the following equation:

(3) $$\begin{array}{c} {\text{RSS}}_{r}=\left(p+1\right)\varepsilon_{x}^{2}. \end{array}$$

The sum of squared residuals of unconstrained regression models can be calculated by the following equation:

(4) $$\begin{array}{c} {\text{RSS}}_{ur}=\left(p+1\right)\varepsilon_{y}^{2}. \end{array}$$

Then, the *F* test was applied to confirm the statistics significant of the residuals:

(5) $$\begin{array}{c} F=\frac{\left({\text{RSS}}_{r}-{\text{RSS}}_{ur}\right)/p}{{\text{RSS}}_{ur}/\left(T-p-q-1\right)}, \end{array}$$

where *T* is the number of sample size which used to estimate *X*. *p* and *q* are the maximum lag number in the regression model. If the signification is confirmed, variable *Y* Granger causes variable *X*.

According to the definition of Granger, the causality coefficient of *Y* on *X* can be calculated as

(6) $$\begin{array}{c} F_{Y⟶X}=\ln\frac{\mathrm{var}\left(\varepsilon_{x}\right)}{\mathrm{var}\left(\varepsilon_{y}\right)}. \end{array}$$

Noted that there exists two kinds of Granger causality interrelations *F*_*X*⟶*Y*_ and *F*_*Y*⟶*X*_, but *F*_*Y*⟶*X*_ ≠ ±*F*_*X*⟶*Y*_.

In this study, EEG signals which were recorded from contralateral premotor, primary motor, and primary sensory area in motor execution and imagery experiment were set as *X* and *Y*, and Granger causality relation was calculated trial by trial.

For example, EEG signal in the premotor area was set as *Y*, and EEG signal in the motor area was set as *X*. If *F*_*Y*⟶*X*_, we noted this relationship as Granger causality from the premotor to motor area.

There are six Granger causality relations between contralateral different brain areas: from premotor to motor, from premotor to sensory, from motor to sensory, from motor to premotor, from sensory to premotor, and from sensory to motor. Because some trials do not have any static significant relation, the number of Granger causality trials was computed. Then, the Granger causality coefficient of significant Granger causality trials was calculated.

## 3. Result

**Figure 7:**
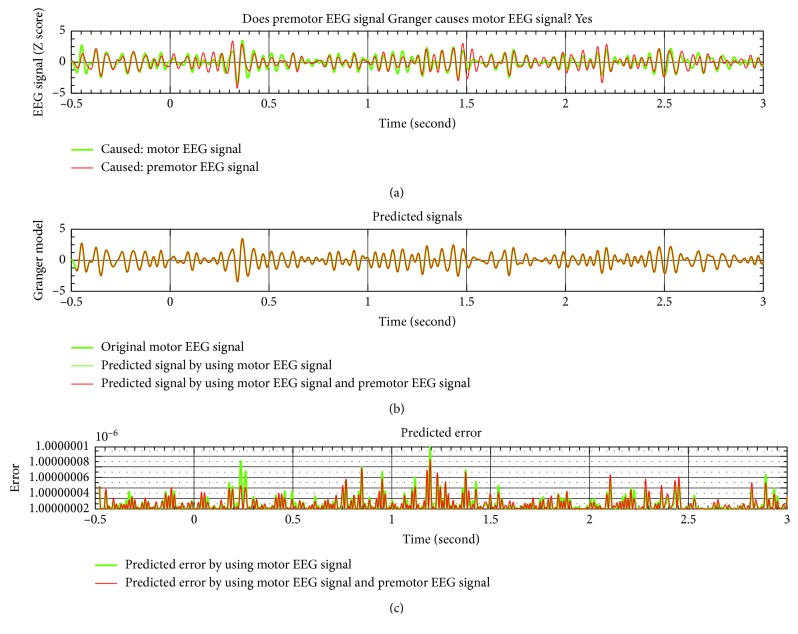
Granger causality result of one finger movement trial.

The G-causality result of one trial from premotor to motor is shown in Figure 7. In Figure 7(a), the beta-band activities of premotor and motor EEG signals are shown. In Figure 7(b), the original motor EEG and predicted signal only from motor EEG and predicted signal from premotor EEG and motor EEG are shown, respectively. From Figure 7(c), the predicted error reduced by using premotor EEG and motor EEG. Thus, significant Granger causality exists in this trial.

The number of trials which has Granger causality relation during left finger execution, left finger imagery, right finger execution, and right finger imagery experiment is shown in Tables 2–5, respectively. The bold number showed the highest number of Granger causality trial between the premotor to motor area. The number of trials from right premotor to motor area was 37 ± 7.3 (mean ± standard deviation), from premotor to sensory area was 29.7 ± 11.3, from motor to sensory area was 27.8 ± 10.6, from motor to premotor area was 23.5 ± 7.9, from sensory to premotor area was 23.4 ± 7.5, and from sensory to motor area was 28.5 ± 12.9 during left finger execution.

A two-way ANOVA is then applied to analyze the number of Granger causality trials between different brain areas. The number of Granger causality from right premotor to right motor area is significantly higher than from right motor to right premotor and from right sensory to right premotor area (*F*_10,5_ = 4.51, *p*=1.8 × 10^−3^), and no other significant differences are observed between the number of Granger causality trials between brain areas during the left finger execution phase.

The number of Granger causality from the right premotor to right motor area is significantly higher than that from the right sensory to right premotor area (*F*_10,5_ = 2.41, *p*=4.92 × 10^*−2*^), and no other significant differences are observed between the number of Granger causality between brain areas during the left finger imagery phase.

No significant differences are observed between the number of Granger causality between the whole brain areas (*F*_10,5_ = 1.99, *p*=9.67 × 10^*−2*^) during the right finger execution phase.

The number of valid Granger causality trials from left premotor to left motor area, left motor to left sensory area, and left premotor to left sensory are significantly higher than that from right sensory to right premotor area (*F*_10,5_ = 11.03, *p*=3.44 × 10^*−7*^), from left motor to left motor area is significantly higher than left motor to left premotor area, and no other significant differences are observed between the number of Granger causality between brain areas during the right finger imagery phase.

The Granger causality coefficient of the Granger causality trial during left finger execution, left finger imagery, right finger execution, and right finger imagery experiment is shown in Tables 6–9, respectively. The bold number showed the lowest Granger causality coefficient between the premotor to motor area. The number of trials from right premotor to motor area was 9.1 ± 3.2, from premotor to sensory area was 13.9 ± 0.6, from motor to sensory area was 13.8 ± 0.7, from motor to premotor area was 14.1 ± 0.5, from sensory to premotor area was 13.6 ± 0.7, and from sensory to motor area was 13.2 ± 0.8 during left finger execution.

A two-way ANOVA is then applied to analyze the Granger causality coefficient between different brain areas. The Granger causality coefficient from right premotor to right motor area is significantly lower than other brain areas (*F*_10,5_ = 23.09, *p*=6.16 × 10^*−12*^), and no other significant Granger causality coefficient differences are observed between brain areas during the left finger execution phase.

The Granger causality coefficient from right premotor to right motor area is significantly lower than other brain areas (*F*_10,5_ = 6.03, *p*=1.93 × 10^*−4*^), and no other significant Granger causality coefficient differences are observed between brain areas during the left finger imagery phase.

No other significant Granger causality coefficient differences are observed between brain areas (*F*_10,5_ = 0.89, *p*=0.49) during the right finger execution phase.

No other significant Granger causality coefficient differences are observed between brain areas (*F*_10,5_ = 0.39, *p*=0.85) during the right finger imagery phase.

## 4. Discussion

The brain areas involved in motor execution and motor imagery were investigated by using fMRI [44–46] and PET [44]. Stephan et al. suggested that imaginative motion activated medial and lateral premotor areas, anterior cingulate areas, and ventral motor anterior areas. The motor execution associated with imaginative movement leads to additional activity in the left primary sensory cortex and premotor area, the premotor cingulate area, and the rostral portion of the left superior parietal cortex [44]. Porro et al. also supports the hypothesis that motor imagery and motor execution are involved in overlapping neural networks in peripheral cortical regions [45]. Ehrsson et al. demonstrated that voluntary motion images of fingers, toes, and tongue activated specific motion representations of corresponding body parts especially in the lateral primary motor cortex [46]. These researches showed that the motor execution and motor imagery of finger movement shared some common brain areas and can be distinguished in premotor areas. Motor imagery may serve as a potential motor training to the rehabilitation of motor control [47] for the patients who suffered from severe upper limb contralateral hemiparesis after brain stroke [48, 49].

In this paper, we investigated the neural mechanism of thirty finger movements in two scenarios: motor execution and motor imagery using the G-causality analysis model. Eleven subjects joined the experiment and performed and imagined left and right finger gestures. EEG signals were recorded simultaneously during motor execution and movement imagery tasks. Beta-band activities of primary motor area, premotor area, and primary sensory area were the most relevant selected feature and then analyzed. We aimed to investigate whether the Granger causality relation can be used for motor function evaluation and motor recovery.

Previous magnetoencephalography studies observed clear movement-related power decreases in the alpha (8–13 Hz) and beta (13–30 Hz) band to 0.5 s during bilateral hand movements in both scenarios: motor execution and motor imagery. In addition, a clear postmovement beta rebound between 0.5 and 1 s was observed when someone moves his/her bilateral hands [50]. Thus, the beta-band EEG signals from −0.5 to 3 seconds of the motor execution and imagery onset were feature selected and analyzed.

We mainly investigated the number of Granger causality trials and Granger causality coefficient between different brain areas during finger motor execution and imagery experiment. The highest number of Granger causality trials 37 ± 7.3, 35.5 ± 8.8, 36.3 ± 10.3, and 39.2 ± 9.0 was achieved from premotor to motor area during left motor execution and imagery and right finger motor execution and imagery, respectively, as shown in Tables 2–5.

These results suggested the most important connection in motor function was from premotor to motor area, which is consistent with previous research on the neuronal population activity model [51]. It suggested Granger causality results were related to motor function and might be used as an efficient feature for motor function evaluation.

Hammer et al. showed the neurons in premotor and motor area activities in the neuronal population level according to the balance of excitatory and inhibitory synaptic input. In addition, significant difference of the Granger causality trial number was observed during left finger execution and imagery and right finger imagery, but not observed during the right finger execution phase.

Meanwhile, the lowest Granger causality coefficients 9.1 ± 3.2, 10.9 ± 3.7, 13.2 ± 0.6, and 13.4 ± 0.6 were achieved from premotor to motor area during left motor execution and imagery and right finger motor execution and imagery, respectively, as shown in Tables 6–9. Significant difference of the Granger causality coefficient was observed during left finger execution and imagery, but not observed during right finger execution and imagery phase.

Several researches showed the significant difference in the brain cortex between left-handed and right-handed subjects. Male right handers showed a significant deeper central sulcus on the left hemisphere than on the right in previous research study [52]. The asymmetry of central sulcus depth is significantly different between left-handed and right-handed individuals in the contralateral hemisphere [53]. In addition, when right-handed subjects performed motor tasks with their right hand, the activation intensity of the cortex in the left hemisphere of the brain was higher than that in the right hemisphere [54–56].

The results in this study also suggested the significant differences between left and right finger motor Granger connection and coefficient may be caused by the reason that all of participants in this study were right-handed. These results also suggested that different MI-based brain motor function recovery strategy should be taken for right-hand subjects and left-hand patients to build an efficient motor recovery protocol after brain stroke. The left-hand subjects should be enrolled to confirm the results in the future. In the future study, we would like to measure the Granger causality in the contralateral motor, motor, and sensorimotor areas of the right and left hemispheres of patients with stroke and then calculate the Granger causality intensity between the brain areas. The efficacy of the motor imagery paradigm might be obtained by the Granger causality model comparing heal people with patients.

There are some limitations in this research. Only beta-band activity of EEG signals was calculated to build the brain network. Compared with ECoG signals, the gamma activity in EEG signals was not so clear and high quality [1, 57]. The alpha band and gamma band were not included although beta-band activity was known to reflect motor function well. Although these areas including the main parts of motor functions are still not enough for free voluntary movement [58, 59], the brain network in present work only focus on left and right sides of the primary motor area, premotor area, and primary sensory area, in totally six points. More areas should be considered, and the brain connectivity should be more investigated in the future work.

## Figures and Tables

**Table 1:** Number of valid trials.

	Left finger execution	Left finger imagery	Right finger execution	Right finger imagery
Subject 1	55	53	58	56
Subject 2	54	50	52	54
Subject 3	56	56	49	53
Subject 4	50	48	54	54
Subject 5	56	58	52	54
Subject 6	46	52	54	50
Subject 7	54	54	56	56
Subject 8	58	58	56	58
Subject 9	53	54	57	55
Subject 10	53	54	53	57
Subject 11	30	44	58	55

**Table 2:** The number of Granger causality trials in left finger execution.

From	To		
	PMA right	MA right	SA right
PMA right	NAN	**37** ± **7.3**	29.7 ± 11.3
MA right	23.5 ± 7.9	NAN	27.8 ± 10.6
SA right	23.4 ± 7.5	28.5 ± 12.9	NAN

**Table 3:** The number of Granger causality trials in left finger imagery.

From	To		
	PMA right	MA right	SA right
PMA right	NAN	**35.5** ± **8.8**	29.3 ± 8.8
MA right	27.1 ± 9.2	NAN	28.3 ± 10.7
SA right	25.5 ± 7.4	31.4 ± 12.4	NAN

**Table 4:** The number of Granger causality trials in right finger execution.

From	To		
	PMA left	MA left	SA left
PMA left	NAN	**36.3** ± **10.3**	34.5 ± 8.2
MA left	24.5 ± 10.9	NAN	30.5 ± 9.3
SA left	25.6 ± 9.6	31.8 ± 9.4	NAN

**Table 5:** The number of Granger causality trials in right finger imagery.

From	To		
	PMA left	MA left	SA left
PMA left	NAN	**39.2** ± **9.0**	36.1 ± 7.7
MA left	24.2 ± 5.7	NAN	33.1 ± 9.1
SA left	22.3 ± 5.6	31.4 ± 7.4	NAN

**Table 6:** Granger causality coefficient of left finger execution.

From	To		
	PMA right	MA right	SA right
PMA right	NAN	**9.1** ± **3.2**	13.9 ± 0.6
MA right	14.1 ± 0.5	NAN	13.8 ± 0.7
SA right	13.6 ± 0.7	13.2 ± 0.8	NAN

**Table 7:** Granger causality coefficient of left finger imagery.

From	To		
	PMA right	MA right	SA right
PMA right	NAN	**10.9** ± **3.7**	14.0 ± 0.6
MA right	14.0 ± 0.4	NAN	13.9 ± 0.7
SA right	13.5 ± 0.9	13.3 ± 0.5	NAN

**Table 8:** Granger causality coefficient of right finger execution.

From	To		
	PMA left	MA left	SA left
PMA left	NAN	**13.2** ± **0.6**	13.4 ± 0.9
MA left	13.6 ± 0.7	NAN	13.6 ± 1.1
SA left	13.7 ± 0.9	13.7 ± 0.6	NAN

**Table 9:** Granger causality coefficient of right finger imagery.

From	To		
	PMA left	MA left	SA left
PMA left	NAN	**13.4** ± **0.6**	13.4 ± 0.8
MA left	13.7 ± 0.8	NAN	13.6 ± 1.0
SA left	13.6 ± 0.8	13.5 ± 0.6	NAN

## Data Availability

The datasets generated and analyzed during the current study are not publicly available due to the policy of Tianjin University of Technology, Capital Medical University, and North China University of Science and Technology, but are available from the corresponding author on reasonable request.
